# 107. Normocephalic Colombian Children with Antenatal Zika Virus Exposure Have Neurodevelopmental Differences at Age 7 Years

**DOI:** 10.1093/ofid/ofaf695.042

**Published:** 2026-01-11

**Authors:** Sarah B Mulkey, Regan Andringa-Seed, Heather Gordish-Dressman, Colleen Peyton, Gilbert Vezina, Michael Msall, Margarita Arroyave-Wessel, Roberta L DeBiasi, Elizabeth Corn, Meagan E Williams, Carlos A Cure, Madison Berl

**Affiliations:** Children's National Hospital/ George Washington University School of Medicine and Health Sciences, Washington, DC; Children's National Hospital, Washington, District of Columbia; Children's National Hospital, Washington, District of Columbia; Northwestern University, Chicago, IL; Children's National Health System, Washington, DC; University of Chicago, Chicago, Illinois; Children's National Hospital, Washington, District of Columbia; Children's National Hospital/The George Washington University School of Medicine and Health Sciences, Washington, District of Columbia; Children's National Hospital, Washington, District of Columbia; Children's National Hospital, Washington, District of Columbia; BIOMELAB SAS, Barranquilla, Atlantico, Colombia; Children's National Hospital, Washington, District of Columbia

## Abstract

**Background:**

The long-term neurodevelopmental outcomes of children with antenatal Zika-virus (ZIKV) exposure who did not have congenital Zika syndrome (CZS) at birth are not well known. In our cohort of Colombian children with antenatal ZIKV exposure and no CZS, cognitive outcomes at age 5 found lower full-scale IQ scores compared to non-exposed controls.

**Methods:**

This prospective case-control study evaluated multidomain developmental outcome trajectory and brain MRI at age 7 years in ZIKV-exposed children without CZS (Cases) and non-exposed children (Controls) from the same communities. We analyzed trends in cognitive scores over time from age 5 to 7 using a mixed effects linear regression model.

**Results:**

46 Cases and 59 Controls were evaluated at mean (SD) ages 7.05 (0.14) and 7.09 (0.13) years, respectively. Cases had lower scores in Wechsler Preschool and Primary Scale of Intelligence verbal comprehension (73.6±8.9 vs 81.3±13.9, p=0.001) and working memory (81.9±9.6 vs 92.6±15.7, p< 0.001). There were no differences between Cases and Controls on motor and receptive vocabulary assessments. Parent-reported adaptive behavior, executive function, and social emotional competencies did not differ between groups, except for the social composite of the Adaptive Behavior Assessment System (Cases vs Controls: 97.7 ± 13.4 vs 92.9 ± 11.7, p=0.048). Mean Case IQ scores, on average, increased between ages 5 (68.49±1.79) and 7 (72.99±1.79) (p=0.026), indicating a positive trajectory. Control children showed no change over time (77.97±1.63 to 75.77±1.6, p=0.22). There was an interaction between groups, indicating differing trajectories (p=0.013). Case MRI findings included white matter injuries (n=6), small cysts (n=4), partially empty sella (n=3), possible polymicrogyria (n=1), mild ventriculomegaly/cerebral atrophy (n=2), sagittal synostosis (n=1), enlarged choroid plexus (n=1), and bilateral vestibular dysplasia (n=1).

**Conclusion:**

ZIKV-exposed children without CZS continue to demonstrate lower cognitive ability compared to controls and some have non-specific MRI findings. Longitudinal follow-up for children with congenital infectious exposures are needed to improve understanding of the full spectrum of school-age outcomes.

**Disclosures:**

All Authors: No reported disclosures

